# The Perception of Minerals and Their Prevalence in Fortified Foods and Supplements in Japan

**DOI:** 10.3390/nu14132586

**Published:** 2022-06-22

**Authors:** Tsuyoshi Chiba, Nanae Tanemura, Chiharu Nishijima

**Affiliations:** Department of Food Function and Labeling, National Institute of Health and Nutrition, National Institutes of Biomedical Innovation, Health and Nutrition, Shinjuku-ku, Tokyo 162-8363, Japan; n-tanemura@nibiohn.go.jp (N.T.); c-nishijima@nibiohn.go.jp (C.N.)

**Keywords:** minerals, salt, fortified food, dietary supplements, nutritional status

## Abstract

People’s intake of some minerals does not meet the nutrient reference values even in high-income countries. Recently, the deficiency of zinc and/or selenium has been considered to cause greater risk of COVID-19 infection and severity. To investigate consumer awareness, we conducted a cross-sectional questionnaire online survey among Japanese people (7500 males and 7500 females) concerning their perceptions of each mineral and the prevalence of mineral-fortified foods and/or mineral supplements. People’s perception of each mineral varied: the highest was for calcium (91.8%) and the lowest was for selenium (44.7%). In addition, only a portion of participants believed that they consumed a sufficient amount of each mineral; the highest was sodium (23.7%), and the lowest was manganese (5.2%). In addition, 18.2% of them felt that they could not consume enough sodium, even though most of the Japanese’s intake is excessive. Among mineral-fortified-food and/or mineral-supplement users, the purposes for these products were to maintain health (80.6%), supplement nutrients (48.0%), and prevent infectious diseases (23.2%). Only 18.4% of participants knew what amount they took. In conclusion, education is needed to prevent not only the insufficiency/deficiency of each mineral but also an excess intake of sodium.

## 1. Introduction

Micronutrients (vitamins and minerals) are essential for the maintenance of health in human beings. Micronutrients play important roles as cofactors or coenzymes and have important functions to neutralize oxidant species and immune systems [1,2]. There are 13 vitamins as essential nutrients—vitamins A, B_1_, B_2_, B_6_, B_12_, C, D, E, K, niacin, pantothenic acid, biotin, and folate. On the other hand, minerals are classified into essential or non-essential. Essential minerals are defined as those whose deficiency in the body is associated with diseases, such as sodium, potassium, calcium, magnesium, zinc, iron, and others. To control the intake of these nutrients to meet the estimated average requirement (EAR), recommended dietary allowance (RDA), or adequate intake (AI) is important. In this regard, a balanced diet is recommended by the World Health Organization (WHO) [3]. Indeed, it has been reported that higher diet-quality scores have been consistently associated with a lower risk of mortality across the world [4,5,6], including in Japan [7]. However, it is difficult to maintain a balanced diet every day in daily life, and some of the nutrients in diets are usually below the EAR. This situation is not only affirmed in Japan but also in other countries. The National Health and Nutrition Examination Survey 2007–2010 in the USA showed that some vitamins (vitamins A, C, D, E, K, and folate) and minerals (magnesium, calcium, and potassium) were under-consumed compared with the EAR in almost all population groups [8]. Among these nutrients, there was a prevalence of over 6% of vitamin B_6_, B_12_, C, and D deficiency in several subpopulations. In Japan, it is reported that skipping breakfast was associated with deficiencies of vitamins A, B_1_, and B_2_ and some minerals among female junior high school students [9]. Another study showed that meals prepared away from home caused a lower intake of vitamin C and some minerals [10]. Consumers should supplement their nutrient intake with dairy foods, but nutrient-fortified foods and/or dietary supplements are also helpful.

In recent decades, the prevalence of dietary supplement use has increased in all generations from infants to elderly persons [11,12,13,14]. Today, there are many ingredients in supplements, not only of vitamin/mineral nature but also herbs and other ingredients derived from plants and animals. However, vitamin/mineral supplements are still the most popular among all generations for the maintenance of health, and an appropriate utilization of vitamin/mineral supplements might complement insufficiency/deficiency of nutrients [11,12,13,14]. Indeed, it has been reported that the inadequacy of vitamin/mineral intake can be improved by vitamin/mineral supplement use [15,16]. In addition, vitamin-/mineral-fortified foods might also complement the insufficiency/deficiency of nutrients [17]. A systematic review reported that vitamin/mineral fortification (in rice and flour; dairy products; beverages; biscuits; spreads; and salt) may reduce anemia by 32% (RR, 0.68; 95% CI, 0.56–0.84); iron deficiency anemia by 72% (RR, 0.28; 95% CI, 0.19–0.39), iron deficiency by 56% (RR, 0.44; 95% CI, 0.32–0.60); vitamin A deficiency by 58% (RR; 0.42, 95% CI, 0.28–0.62); vitamin B_2_ deficiency by 64% (RR, 0.36; 95% CI, 0.19–0.68); vitamin B_6_ deficiency by 91% (RR, 0.09; 95% CI, 0.02–0.38); and vitamin B_12_ deficiency by 58% (RR, 0.42; 95% CI, 0.25–0.71) [18]. These data suggest that fortified foods may improve our health, even though this is low-quality evidence because of study limitations, imprecision, high heterogeneity, and small sample size. In Japan, consumers can buy several vitamin-/mineral-fortified foods at supermarkets, online markets, pharmacies, or other places. Besides regular foods, such as milk, yogurt, cereal, etc., vitamin-/mineral-fortified foods can be incorporated in a regular diet.

Previously, we reported the perception of vitamins and their prevalence in fortified foods and dietary supplements in Japan [19]. The highest perception was for vitamin C (93.2%), but only 22.3% of participants believed that they took sufficient amounts of vitamin C. The reason why most participants did not use vitamin-fortified foods and/or vitamin supplements was the economic issue. On the other hand, among vitamin-fortified-food and/or vitamin supplement users, the purposes of these products’ usage were varied, such as maintaining health, supplementing nutrients, beauty-related purposes, and preventing infectious diseases. A similar scenario might present with minerals. Therefore, we conducted a cross-sectional questionnaire online survey to clarify the knowledge and awareness of mineral intake among consumers and to investigate whether consumers used mineral-fortified foods/mineral supplements and whether they were aware of their intake levels of the 13 minerals (sodium, potassium, calcium, magnesium, phosphorus, zinc, iron, copper, manganese, iodine, selenium, chromium, and molybdenum) that are listed in the dietary reference intake for Japanese people (DRI-J).

## 2. Materials and Methods

### 2.1. Participants and Procedures

A cross-sectional questionnaire online survey was conducted by Cross Marketing Inc. (Tokyo, Japan) from 8 to 11 November 2021. The questionnaire consisted of 2 stages. First, preliminary survey was conducted on 15,000 respondents aged 20–79 years old comprised of 7500 males and 7500 females. The preliminary survey contains following questions about the usual dietary status, mineral-fortified foods or/and mineral supplement usage status, reasons that they did not use mineral-fortified foods or/and mineral supplement, and awareness of each mineral and intake status. Then, additional survey was conducted on 2077 respondents (1032 males and 1045 females) who used mineral-fortified foods and/or mineral supplements in preliminary survey. The additional survey contains following questions about purpose of mineral-fortified foods or/and mineral supplement use, knowledge about the DRI-J, awareness of each mineral intake, and application program usage status. This study was in accordance with the Declaration of Helsinki, and approved by the Research Ethics Committee of the National Institutes of Biomedical Innovation, Health and Nutrition (No. 297; 15 September 2021). Questionnaire was modified our previous questionnaire conducted on vitamins [19] (supplementary file: File S1). Detailed procedures of an internet survey are described in our previous report [19].

### 2.2. Statistical Analysis

Differences in distribution among groups were compared using the chi-squared (χ^2^) test. All statistical analyses were performed using Cross finder 2 ver.2.3.2.0 that was provided by Cross Marketing Inc. (Tokyo, Japan). *p*-value of <0.05 was considered statistically significant.

## 3. Results

### 3.1. Characteristics

The 15,000 respondents (49.4 ± 16.5 years old) comprised of 7500 males (49.6 ± 16.5 years old) and 7500 females (49.3 ± 16.6 years old). Their educational backgrounds were as follows: high school, 5279 (35.2%); college/university, 8902 (59.3%); graduate school, 578 (3.9%); other, 241 (1.6%). The distribution of healthcare professionals was as follows: doctors, 140 (0.9%); pharmacists, 182 (1.2%); dieticians, 307 (2.0%).

### 3.2. Well-Balanced Diet

The definition of a well-balanced diet in this study is having a meal consisting of the following three dishes at least twice a day. 1. Grain dish (rice, bread, noodles, etc.), 2. Main dish (dishes mainly made of meat, fish, eggs, soybeans, soy products, etc.), 3. Side dish (dishes mainly made of vegetables, seaweed, mushrooms, etc.). Regarding, the frequency of consuming a well-balanced diet, 35.2% of participants answered “almost every day”, and 20.7% of them answered “4–5 days a week”, while 25.1% of them answered “Rarely” (Table 1). Then, we asked the participants who answered “rarely” why they could not follow a well-balanced diet; 41.5% of the participants answered “I have no time to spare”, and 36.3% of them answered “I do not have enough money”. This result is almost the same as that of the previous study [19].

### 3.3. The Prevalence of Mineral-Fortified Food and Mineral Supplement Usage

The prevalence of mineral-fortified food usage is shown in Table 2. Only 7.9% of them answered “I actively use it” and 24.8% answered “I have never used it”. The main reason for answering “I never used it” was “I feel that I can take enough with my usual diet” (32.1%), followed by “It is expensive to buy” (28.1%) and “I do not want to spend money on fortified foods” (24.1%). The prevalence of mineral-fortified food usage was associated with the awareness of usual diets. A greater prevalence of mineral-fortified food usage was in participants who usually consumed a well-balanced diet, which is presented compared to participants who did not (Table 3).

The prevalence of mineral supplement usage is shown in Table 4. Only 9.3% of them answered “I actively use mineral supplement” and 59.5% answered “I have never used it”. The main reason for answering “I have never used it” was “I do not want to spend money on supplements” (55.5%), followed by “It is expensive to buy” (23.3%) and “I feel that I can take it enough with my usual diet” (22.8%). Similar to mineral-fortified foods, the prevalence of mineral supplement usage was also associated with the awareness of usual diets. A greater prevalence of mineral supplement usage in participants who usually consumed a well-balanced diet was presented compared to participants who did not (Table 5).

Users of both fortified foods (“I actively use it” and “I use it occasionally”) and supplements (“I currently use mineral supplement”) comprised 6.9% of the participants.

### 3.4. The Prevalence of “Foods with Nutrient Function Claims”

“Foods with Nutrient Function Claims” is one of the food categories regulated by the Japanese government. When asked about the prevalence of “Foods with Nutrient Function Claims”, 16.6% of them answered “I currently use it” (Table 6). “Foods with Nutrient Function Claims” contain not only minerals, but also vitamins and n-3 fatty acid. In addition, “Foods with Nutrient Function Claims” have the form of not only capsules, tablets, and powders but also regular food. In this regard, the prevalence was higher than mineral supplements.

### 3.5. The Perception of Minerals

To clarify how degree consumers know about each mineral, we asked participants about the perception of each mineral from “I understand its role in the body well” to “I do not know”. The percentages of respondents who answered “I understand its role in the body well” were the highest for calcium (24.9%), then iron (19.1%) and potassium (12.1%) and were the lowest for molybdenum (3.8%) (Table 7). Perception was the highest for calcium (91.8%), and 7 of 13 minerals were more than 80%. On the other hand, perception of selenium and molybdenum were less than 50%.

### 3.6. Awareness of One’s Mineral Intake

To clarify how degree consumers recognized that they were taking each mineral by themselves, we asked participants about the awareness of their mineral intake status from “Enough” to “I do not know”. The minerals ranked by the proportions of the answer “Enough” were sodium (23.7%) in first place, followed by calcium (22.5%) and potassium (16.3%), and 7 of 13 minerals were less than 10% (Table 8). Regarding the answer “Insufficient”, calcium (31.0%) ranked first, and sodium (18.2%) was last. However, more than half of them answered “I do not know” for all the minerals except for calcium (46.5%).

### 3.7. Conscious Intake of Minerals

To clarify which minerals consumers were consciously ingesting, we asked participants to choose minerals that they consciously intake. Similar to their perception of each mineral, the participants consciously ingested calcium (32.6%), iron (20.5%), potassium (12.1%), and zinc (11.3%) (Table 9). However, 9 of 13 minerals were taken by almost less than 10%. A gender difference was recognized in conscious mineral intake, and females were more conscious than males in terms of potassium, calcium, magnesium, and iron intake. On the other hand, males were more conscious than females in terms of sodium, phosphorus, zinc, copper, manganese, and selenium intake.

We conducted an additional questionnaire on mineral-fortified-food users (who answered “I actively use it” in Table 2) and/or mineral supplement users (who answered “I currently use mineral supplement” in Table 4) (*n* = 2077; 1032 males and 1045 females). The most consciously ingested mineral was calcium (60.9%), then iron (46.7%) and zinc (37.1%) (Table 10). These are 2–3 times those in all participants. Females were more conscious than males in terms of calcium and iron intake. On the other hand, males were more conscious than females in terms of sodium, magnesium, phosphorus, zinc, copper, and manganese intake.

### 3.8. Purpose of Use of Mineral-Fortified Foods and Mineral Supplements

The primary purpose of mineral-fortified food/mineral supplement use was “Maintenance of health” (80.6%), followed by “Supplementation of nutrients” (48.0%) (Table 11). Even though it is not allowed for any other health claim, some participants used mineral-fortified food/mineral supplements for “Prevention of diseases” (25.6%), “Beauty benefits” (18.2%) and “Improvements to health” (18.2%). Gender differences were recognized in regard to the purpose of using these products: “Beauty benefits”, “Weight loss” and “Improvements to health” were higher in females than in males; on the other hand, “Maintenance of health” and “Building muscle” were higher in males than in females.

### 3.9. Utilization of the Dietary Reference Intakes for Japanese People, Food Labeling on Products, and Application Programs

Among mineral-fortified-food/mineral supplement users, only 10.6% of the participants used the DRI-J (Table 12). Most of the participants answered “I have only heard about it” or “I do not know about it”. There were no gender differences of perception and utilization of DRI-J.

The amount of mineral content is labeled on mineral-fortified foods/mineral supplements. Labeling is the capital tool to recognize the nutrient contents in products. However, only 30.7% of mineral-fortified-food/mineral supplement users always checked it, and 47.3% of them sometimes checked it (Table 13). Surprisingly, 3.8% of mineral-fortified food/mineral supplement users did not know that mineral contents were labeled on packages. A slight gender difference was recognized in regard to the confirmation of labeling. According to the checking of nutrition labels, only 14.0% of participants checked the amount of each mineral they were taking, while 61.1% did not check it, and 24.9% did not care about it (data not shown).

Consumers can know the amount of each mineral that they take by checking the labels on mineral-fortified foods/mineral supplements. However, consumers also take minerals from general foods. These days, many application programs for smartphones that can analyze nutrient contents from pictures are available. It makes us easy to recognize the amount of nutrients, including minerals, that we take from foods. However, only 10.9% the participants used these application programs (data not shown). In this situation, only 18.4% of participants knew their mineral intake amount (Table 14). There were gender differences in the knowledge about the amount of mineral intake, with males scoring higher than females.

## 4. Discussion

In this study, the perception and the prevalence of each mineral showed varying results among minerals. In addition, most of the consumers, including mineral-fortified-food/mineral supplement users, were not aware of the nutritional status of each mineral themselves. In Japan, the labeling of minerals, except sodium, on product packages is voluntary. So, even though consumers check food labels, they cannot know how much of a certain mineral is contained in each food.

In this regard, a well-balanced diet is recommended to avoid deficiency/insufficiency of specific nutrients, including minerals. It has been reported that a well-balanced diet decreases the risk of mortality [4,5,6,7]. Recently, it is also reported that well-balanced meals for lunch could reduce the risks of lifestyle-related diseases in working men [20]. In addition, a well-balanced diet is also encouraged to prevent COVID-19 [21,22]. However, only one-third of participants consumed a well-balanced diet almost every day in this study, and this result is almost the same as that of our previous study (33.2%) [19]. The major reasons why participants could not consume a well-balanced diet were time and money. It is an important issue for the Japanese government to resolve to achieve the Sustainable Development Goals.

At this time, the inadequacy of the intake of some minerals has been reported in Japan [10] and other countries [15,23,24]. On the other hand, vitamin-/mineral-fortified-food/supplement use could improve the inadequate intake of these nutrients [15,16,17,23]. However, with vitamin/mineral supplement use, there is also a risk of excess intake of these nutrients, and the intake of some of them is greater than upper limit [25,26], especially children [27,28]. In Japan, there is no regulation of the amount of vitamin/mineral content in each product except for “Foods with Nutrient Function Claims”. In this situation, the daily recommended dose of some vitamins/minerals is already over its UL in some products on the market. However, awareness of mineral intake was very low in our participants and some of them did not check the label on the dietary supplements that they used. In addition, consumers also intake these vitamins/minerals from daily foods. To avoid excess intake, consumers should be aware not only of their actual intake of each vitamin/mineral from daily foods but also any additional amount from dietary supplements. We did not include children in this study, but more attention needs to be paid to this generation [29].

Labels on food products are helpful to recognize the amount of nutrient intake. Many consumers were aware of them and checked them. However, the consumption of each ingredient was not significantly different between label users and non-users [30]. This means that consumers may lack nutritional knowledge to choose beneficial products for their health. Indeed, some participants in our study answered that they consciously ingested sodium, even though the suppression of sodium intake is one of the priority issues in Japan. The National Health and Nutrition Survey Japan showed that the Japanese consumed about 10 g salt/day (11.0 g in males, 9.3 g in females) in 2018, which is twice the amount (5 g/day) recommended by the WHO [31]. High intake of sodium was the leading dietary risk of death and DALYs in Japan, even though Japan had the lowest rate of all diet-related deaths and DALYs [32]. It was reported that about 90% of Japanese consumers (high school students and elderly people) recognized that excess salt intake was the cause of hypertension [33]. However, 70% of high school students and 89% of elderly people could not link sodium to salt. In addition to these reports, 11.0% of participants did not know sodium, and 22.3% of participants answered that they consciously took sodium in this study. So, the Japanese government made the labeling of sodium content as salt equivalent in nutrient tables mandatory. In addition, potassium is also an important mineral that regulates blood pressure, and it seems that the sodium-to-potassium ratio is more significant than either sodium or potassium alone [34,35]. In this regard, potassium contents should also be considered as a priority element in the nutrient profiling and labeling of foods [36].

Nutrient profiling and labeling contain important information for consumers to choose healthy foods and products. At this time, the Codex Committee on Nutrition and Foods for Special Dietary Uses (CCNFSDU), one of the Codex Alimentarius General Subject Committees, has just started to work for the developing of guidelines for nutrient profiles, including front-of-pack labeling (FOPL) as an easy-to-understand display. While international consistency is important, flexibility is also important, because of different situations in each country. As mentioned above, Japan has the highest salt intake in the world; therefore, reducing salt intake and/or increasing potassium intake are/is an important issue(s) [34,35,37,38]. The efficacy of information depends on the consistency between the target population and the contents of the message. However, there are few studies on whether nutrition labels could help consumers make better decisions at the point of purchase [39]. Recently, a meta-analysis was conducted on the efficacy of FOPL [40]. In this review, the FOPL of calories, sugar, saturated fat, and sodium encouraged healthier food purchasing. However, there are still few studies about the efficacy of FOPL for consumption. At this time, there is not enough evidence that FOPL promotes nutrient intake including minerals in the real world, but FOPL might be helpful.

COVID-19 is still one of the most important issues in the world. At this time, anti- SARS-CoV-2 vaccines and drugs are available, but the number of infected patients has increased in the world [41]. Therefore, it is important to strengthen the immune system against SARS-CoV-2 infection [42,43]. It is well-known that malnutrition attenuates the immune system and might be a risk of infection and exaggeration of COVID-19; especially, the association with deficit of vitamin C, vitamin D, zinc, and selenium has been thoroughly studied [44,45]. In the case of each mineral, deficiency or lower plasma levels of zinc [46,47], calcium [47], and selenium [48,49] in COVID-19 patients was reported. In this situation, some dietary supplements on the market that contain these nutrients are claimed to have anti-COVID-19 effects, even though there is no evidence that dietary supplements can prevent COVID-19 in healthy people. However, it has been reported that micronutrients play an important role in the immune response of SARS-CoV-2 vaccination [50]. Therefore, the awareness of these micronutrients in consumers is important to maintain their health and keep their vaccination effective.

The strength of this study is that this is the first report that clarifies the perception of each mineral and its prevalence in fortified foods and supplements in 15,000 participants. In addition, there are some questions that were also asked in our previous study, with the results being almost the same. This confirms and strengthens our results. On the other hand, there are some limitations. We did not survey the actual amount of mineral intake in our participants. Therefore, we could not presume the influence of their awareness on their consumption. Moreover, some participants answered that they could not intake enough sodium and consciously took it, but we did not ask them whether they knew that sodium means salt or not. We should have clarified it, because sodium content in foods is labeled as salt in Japan. This study was conducted using an online survey, so the participants were registrants of the survey company. So, we have to carefully treat our data as general, even though online surveys have become popular across all age groups.

## 5. Conclusions

In this study, the prevalence of mineral-fortified-food usage was 7.9% and for mineral supplement usage it was 9.3%. This prevalence was greater in participants who usually consumed a well-balanced diet. The major purposes of these products’ use were for maintaining health (80.6%) and for the supplementation of nutrients (48.0%). However, most participants did not use mineral-fortified foods and/or mineral supplements because of economic issues. To keep healthy nutritional status of individuals, it is important to improve not only consumer awareness but also their environment, which make consumers to choose a healthier product without imposing any economic burden. In addition, to advocate the policy regarding empowering the consumers with the healthy awareness of mineral intake is also suggested.

## Figures and Tables

**Table 1:** Frequency of a well-balanced diet and reasons for its lack.

	*n*	%
Almost every day	5285	35.2
4–5 days a week	3110	20.7
2–3 days a week	2845	19.0
Rarely	3760	25.1
Reasons for “Rarely” (*n* = 3760) ^1^		
I have no time to spare	1561	41.5
I do not have enough money	1366	36.3
There are many opportunities to eat out	412	11.0
I often buy lunch boxes and prepared dishes	1103	29.3
Others	375	10.0

^1^ Multiple answers.

**Table 2:** The prevalence of mineral-fortified food usage and reasons for non-usage.

	*n*	%
I actively use it.	1179	7.9
I use it occasionally.	3367	22.4
I have never used it.	3724	24.8
I do not care.	6730	44.9
Reasons for not using (*n* = 3724) ^1^		
I feel that I can take enough with my usual diet.	1197	32.1
No mineral-fortified foods that I want to consume.	393	10.6
I use other products such as supplements.	316	8.5
It is expensive to buy.	1048	28.1
I do not want to spend money on fortified foods.	897	24.1
I have bought it, but it was not delicious.	135	3.6
I have bought it, but it did not suit my health.	170	4.6
Others	169	4.5

^1^ Multiple answers.

**Table 3:** The relationship between frequency of a well-balanced diet and prevalence of mineral-fortified food usage.

	*n*	I Actively Use It	I Use It Occasionally	I Have Never Used It	I Do Not Care	*p*-Value
Almost every day	5285	14.2	25.3	24.6	35.9	<0.001
4–5 days a week	3110	7.7	32.0	25.4	34.9	
2–3 days a week	2845	3.7	24.3	28.9	43.2	
Rarely	3760	2.2	9.1	21.7	67.0	

The difference among groups was examined using the chi-squared (χ^2^) test.

**Table 4:** The prevalence of mineral supplement usage and reasons for non-usage.

	*n*	%
I currently use mineral supplement.	1392	9.3
I currently use non-mineral supplement.	3088	20.6
I used to use it, but not now.	2052	13.7
I have never used it.	8926	59.5
Reasons for not using (*n* = 8926) ^1^		
I feel that I can take enough with my usual diet.	2500	22.8
No mineral supplements that I want to consume.	680	6.2
I use other products such as fortified foods.	295	2.7
It is expensive to buy.	2554	23.3
I do not want to spend money on supplements.	6091	55.5
I have bought it but it did not suit my health.	237	2.2
Others	473	4.3

^1^ Multiple answers.

**Table 5:** The relationship between frequency of a well-balanced diet and prevalence of mineral supplement usage.

	*n*	I Currently Use Mineral Supplement	I Currently Use Non-Mineral Supplement	I Used to Use It, But Not Now	I Have Never Used It
Almost every day	5285	12.3	22.3	12.8	56.4
4–5 days a week	3110	11.0	27.6	14.7	51.0
2–3 days a week	2845	7.9	21.0	17.8	55.6
Rarely	3760	4.6	12.1	11.0	73.8

Multiple answers.

**Table 6:** The prevalence of “Foods with Nutrient Function Claims”.

	*n*	%
I do not know about it.	4799	32.0
I know it, but I do not use it.	7716	51.4
I currently use it.	2485	16.6

**Table 7:** The perception of each mineral.

	I Understand Its Role in the Body Well	I Understand Somewhat	I Have Only Heard about It	I Do Not Know	Perception ^1^	*p*-Value
Sodium	11.4	39.0	38.6	11.0	89.0	<0.001
Potassium	12.1	38.0	39.9	10.1	89.9	
Calcium	24.9	41.4	25.5	8.2	91.8	
Magnesium	11.4	41.0	38.0	9.6	90.4	
Phosphorus	6.9	34.6	45.1	13.4	86.6	
Zinc	11.8	37.9	39.5	10.9	89.1	
Iron	19.1	41.5	30.4	9.0	91.0	
Copper	5.6	33.5	45.3	15.7	84.3	
Manganese	4.8	28.9	45.9	20.4	79.6	
Iodine	6.9	30.5	45.0	17.6	82.4	
Selenium	4.4	15.2	25.2	55.3	44.7	
Chromium	4.3	17.1	32.2	46.4	53.6	
Molybdenum	3.8	15.1	26.9	54.2	45.8	

*n* = 15,000, results are shown as percentages (%). ^1^ Perception includes from “I understand its role in the body well” to “I have only heard about it”. The difference among minerals was examined using the chi-squared (χ^2^) test.

**Table 8:** The perception of one’s mineral intake status.

	Enough	Insufficient	I Do Not Know	*p*-Value
Sodium	23.7	18.2	58.2	<0.001
Potassium	16.3	23.1	60.6	
Calcium	22.5	31.0	46.5	
Magnesium	11.2	25.2	63.6	
Phosphorus	8.1	21.8	70.1	
Zinc	10.1	25.8	64.1	
Iron	14.3	30.3	55.5	
Copper	6.0	21.2	72.8	
Manganese	5.8	19.5	74.7	
Iodine	7.3	19.9	72.8	
Selenium	7.7	21.6	70.7	
Chromium	6.4	20.4	73.2	
Molybdenum	6.7	21.1	72.2	

*n* = 15,000, results are shown as percentages (%). The difference among minerals was examined using the chi-squared (χ^2^) test.

**Table 9:** Consciously ingested minerals.

	All	Male	Female
Sodium	7.0	8.1	5.8
Potassium	12.1	11.0	13.1
Calcium	32.6	25.9	39.0
Magnesium	9.8	9.5	10.1
Phosphorus	4.9	5.5	4.3
Zinc	11.3	12.3	10.3
Iron	20.5	14.4	26.5
Copper	4.3	5.0	3.6
Manganese	4.0	4.6	3.5
Iodine	5.4	5.3	5.5
Selenium	6.3	6.6	5.9
Chromium	5.3	5.6	4.9
Molybdenum	5.4	5.2	5.8

*n* = 15,000 (7500 males and 7500 females), results are shown as percentages (%).

**Table 10:** Consciously ingested minerals among mineral-fortified-food/mineral-supplement users.

	All	Male	Female
Sodium	22.3	26.9	17.9
Potassium	33.0	33.6	32.4
Calcium	60.9	55.9	65.8
Magnesium	33.7	34.0	33.4
Phosphorus	17.7	20.2	15.2
Zinc	37.1	41.8	32.4
Iron	46.7	39.6	53.7
Copper	15.3	17.2	13.5
Manganese	14.9	17.0	12.8
Iodine	18.1	18.5	17.8
Selenium	18.7	20.4	16.6
Chromium	15.8	16.6	14.8
Molybdenum	17.0	16.8	17.3

*n* = 2077 (1032 males and 1045 females), Results are shown as percentages (%).

**Table 11:** Purpose of use of mineral-fortified food/mineral supplements.

	All	Male	Female
Maintenance of health	80.6	83.3	77.9
Supplementation of nutrients	48.0	48.3	47.7
Beauty benefits	18.2	11.0	25.3
Weight loss	13.4	11.6	15.1
Building muscle	14.0	16.0	12.1
Improvements to health	18.2	16.4	19.9
Prevention of diseases	25.6	24.0	27.2
Treatment of diseases	5.8	6.0	5.6
Improvement of immune function/prevention of infectious diseases	21.9	20.3	23.5
Other	1.0	0.6	1.4

*n* = 2077 (1032 males and 1045 females), results are shown as percentages (%).

**Table 12:** Perception and utilization of the dietary reference intake for Japanese people.

	All	Male	Female	*p*-Value
I know about it and I use it.	10.6	11.3	9.9	0.072
I know about it, but I do not use it.	15.9	14.6	17.2	
I have only heard about it.	46.3	44.7	47.8	
I do not know about it.	27.2	29.4	25.1	

*n* = 2077 (1032 males and 1045 females), results are shown as percentages (%). The difference between males and females was examined using the chi-squared (χ^2^) test.

**Table 13:** Confirmation of labeling of mineral contents on fortified food/supplements.

	All	Male	Female	*p*-Value
I always check it.	30.7	32.8	28.5	0.025
I sometimes check it.	47.3	47.2	47.5	
I do not check it.	18.2	15.9	20.6	
I did not know that it was labeled.	3.8	4.1	3.4	

*n* = 2077 (1032 males and 1045 females), results are shown as percentages (%). The difference between males and females was examined using the chi-squared (χ^2^) test.

**Table 14:** The knowledge about the amount of mineral intake.

	All	Male	Female	*p*-Value
I know the intake amount.	18.4	23.0	14.0	<0.001
I do not know the intake amount.	60.4	56.0	64.7	
I do not care about it.	21.2	21.0	21.3	

*n* = 2077 (1032 males and 1045 females), results are shown as percentages (%). The difference between males and females was examined using the chi-squared (χ^2^) test.

## Data Availability

The data presented in this study are available upon request from the corresponding author.

## Supplementary Materials

The following supporting information can be downloaded at: https://www.mdpi.com/article/10.3390/nu14132586/s1, File S1: Questionnaire

## References

[1] Evans P., Halliwell B. (2001). Micronutrients: Oxidant/antioxidant status. Br. J. Nutr..

[2] Weyh C., Krüger K., Peeling P., Castell L. (2022). The Role of Minerals in the Optimal Functioning of the Immune System. Nutrients.

[3] World Health Organization Healty Diet. https://apps.who.int/iris/bitstream/handle/10665/325828/EMROPUB_2019_en_23536.pdf?sequence=1&isAllowed=y.

[4] Morze J., Danielewicz A., Hoffmann G., Schwingshackl L. (2020). Diet Quality as Assessed by the Healthy Eating Index, Alternate Healthy Eating Index, Dietary Approaches to Stop Hypertension Score, and Health Outcomes: A Second Update of a Systematic Review and Meta-Analysis of Cohort Studies. J. Acad. Nutr. Diet..

[5] Schwingshackl L., Bogensberger B., Hoffmann G. (2018). Diet Quality as Assessed by the Healthy Eating Index, Alternate Healthy Eating Index, Dietary Approaches to Stop Hypertension Score, and Health Outcomes: An Updated Systematic Review and Meta-Analysis of Cohort Studies. J. Acad. Nutr. Diet..

[6] Schwingshackl L., Hoffmann G. (2015). Diet Quality as Assessed by the Healthy Eating Index, the Alternate Healthy Eating Index, the Dietary Approaches to Stop Hypertension Score, and Health Outcomes: A Systematic Review and Meta-Analysis of Cohort Studies. J. Acad. Nutr. Diet..

[7] Kurotani K., Akter S., Kashino I., Goto A., Mizoue T., Noda M., Sasazuki S., Sawada N., Tsugane S., Japan Public Health Center based Prospective Study Group (2016). Quality of diet and mortality among Japanese men and women: Japan Public Health Center based prospective study. BMJ.

[8] Bruins M.J., Bird J.K., Aebischer C.P., Eggersdorfer M. (2018). Considerations for Secondary Prevention of Nutritional Deficiencies in High-Risk Groups in High-Income Countries. Nutrients.

[9] Matsumoto M., Hatamoto Y., Sakamoto A., Masumoto A., Ikemoto S. (2020). Breakfast skipping is related to inadequacy of vitamin and mineral intakes among Japanese female junior high school students: A cross-sectional study. J. Nutr. Sci..

[10] Matsumoto M., Saito A., Okada C., Okada E., Tajima R., Takimoto H. (2021). Consumption of meals prepared away from home is associated with inadequacy of dietary fiber, vitamin C and mineral intake among Japanese adults: Analysis from the 2015 National Health and Nutrition Survey. Nutr. J..

[11] Chiba T., Sato Y., Nakanishi T., Yokotani K., Suzuki S., Umegaki K. (2014). Inappropriate Usage of Dietary Supplements in Patients by Miscommunication with Physicians in Japan. Nutrients.

[12] Kobayashi E., Nishijima C., Sato Y., Umegaki K., Chiba T. (2018). The Prevalence of Dietary Supplement Use Among Elementary, Junior High, and High School Students: A Nationwide Survey in Japan. Nutrients.

[13] Kobayashi E., Sato Y., Umegaki K., Chiba T. (2017). The Prevalence of Dietary Supplement Use among College Students: A Nationwide Survey in Japan. Nutrients.

[14] Sato Y., Yamagishi A., Hashimoto Y., Virgona N., Hoshiyama Y., Umegaki K. (2009). Use of dietary supplements among preschool children in Japan. J. Nutr. Sci. Vitaminol..

[15] Shakur Y.A., Tarasuk V., Corey P., O’Connor D. (2012). A Comparison of Micronutrient Inadequacy and Risk of High Micronutrient Intakes among Vitamin and Mineral Supplement Users and Nonusers in Canada. J. Nutr..

[16] Murphy S.P., White K.K., Park S.-Y., Sharma S. (2007). Multivitamin-multimineral supplements’ effect on total nutrient intake. Am. J. Clin. Nutr..

[17] Fulgoni V.L., Buckley R.B. (2015). The Contribution of Fortified Ready-to-Eat Cereal to Vitamin and Mineral Intake in the U.S. Population, NHANES 2007–2010. Nutrients.

[18] Das J.K., Salam A.R., Bin Mahmood S., Moin A., Kumar R., Mukhtar K., Lassi Z.S., Bhutta A.Z. (2019). Food fortification with multiple micronutrients: Impact on health outcomes in general population. Cochrane Database Syst. Rev..

[19] Chiba T., Tanemura N., Nishijima C. (2021). The Perception of Vitamins and Their Prevalence in Fortified Food and Supplements in Japan. Nutrients.

[20] Mori M. (2021). Well-Balanced Lunch Reduces Risk of Lifestyle-Related Diseases in Middle-Aged Japanese Working Men. Nutrients.

[21] Jayawardena R., Misra A. (2020). Balanced diet is a major casualty in COVID-19. Diabetes Metab. Syndr. Clin. Res. Rev..

[22] World Health Organization Nutrition Advice for Adults during the COVID-19 Outbreak. http://www.emro.who.int/nutrition/news/nutrition-advice-for-adults-during-the-covid-19-outbreak.html.

[23] Wallace T.C., Frankenfeld C.L., Frei B., Shah A.V., Yu C.-R., Van Klinken B.J.-W., Adeleke M. (2019). Multivitamin/Multimineral Supplement Use is Associated with Increased Micronutrient Intakes and Biomarkers and Decreased Prevalence of Inadequacies and Deficiencies in Middle-Aged and Older Adults in the United States. J. Nutr. Gerontol. Geriatr..

[24] Mensink G.B.M., Fletcher R., Gurinović M., Huybrechts I., Lafay L., Serra-Majem L., Szponar L., Tetens I., Verkaik-Kloosterman J., Baka A. (2013). Mapping low intake of micronutrients across Europe. Br. J. Nutr..

[25] Stoś K., Woźniak A., Rychlik E., Ziółkowska I., Głowala A., Ołtarzewski M. (2021). Assessment of Food Supplement Consumption in Polish Population of Adults. Front. Nutr..

[26] Cowan A.E., Jun S., Tooze J.A., Eicher-Miller H.A., Dodd K.W., Gahche J.J., Guenther P.M., Dwyer J.T., Potischman N., Bhadra A. (2019). Total Usual Micronutrient Intakes Compared to the Dietary Reference Intakes among U.S. Adults by Food Security Status. Nutrients.

[27] Samuel L., Ethan D., Basch C., Dunne S., Quinn C. (2022). An analysis of nutrient facts labels of pediatric multi-vitamin and mineral supplements: Is there a risk of overexposure?. Nutr. Health.

[28] Sicińska E., Pietruszka B., Januszko O., Jakubowski S., Kielak-Biskupska K., Rolf K., Kaluza J. (2020). Intake of Vitamins and Minerals From Voluntarily Fortified Foods and/or Dietary Supplements in School Adolescents in Central-Eastern Poland. Front. Public Health.

[29] Dwyer J.T., Saldanha L.G., Bailen R.A., Gahche J.J., Potischman N., Bailey R.L., Jun S., Long Y., Connor E., Andrews K.W. (2021). Do Multivitamin/Mineral Dietary Supplements for Young Children Fill Critical Nutrient Gaps?. J. Acad. Nutr. Diet..

[30] Anastasiou K., Miller M., Dickinson K. (2019). The relationship between food label use and dietary intake in adults: A systematic review. Appetite.

[31] WHO Guideline: Sodium Intake for Adults and Children. https://www.who.int/publications/i/item/9789241504836.

[32] Afshin A., Sur P.J., Fay K.A., Cornaby L., Ferrara G., Salama J.S., Mullany E.C., Abate K.H., Abbafati C., Abebe Z. (2019). Health effects of dietary risks in 195 countries, 1990–2017: A systematic analysis for the Global Burden of Disease Study 2017. Lancet.

[33] Sanagawa A., Ogasawara M., Kusahara Y., Yasumoto M., Iwaki S., Fujii S. (2017). Investigation into Differences in Level of Knowledge about Hypertension between High School Students and Elderly People. Yakugaku Zasshi.

[34] Filippini T., Violi F., D’Amico R., Vinceti M. (2017). The effect of potassium supplementation on blood pressure in hypertensive subjects: A systematic review and meta-analysis. Int. J. Cardiol..

[35] Perez V., Chang E.T. (2014). Sodium-to-Potassium Ratio and Blood Pressure, Hypertension, and Related Factors. Adv. Nutr. Int. Rev. J..

[36] Jacobson M.F., Campbell N.R.C. (2019). Potassium labeling of foods: Potential benefit for blood pressure. J. Clin. Hypertens..

[37] Okuda N., Okayama A., Miura K., Yoshita K., Saito S., Nakagawa H., Sakata K., Miyagawa N., Chan Q., Elliott P. (2017). Food sources of dietary sodium in the Japanese adult population: The international study of macro-/micronutrients and blood pressure (INTERMAP). Eur. J. Nutr..

[38] Bhat S., Marklund M., Henry M.E., Appel L.J., Croft K.D., Neal B., Wu J.H.Y. (2020). A Systematic Review of the Sources of Dietary Salt Around the World. Adv. Nutr..

[39] Grunert K.G., Wills J.M. (2007). A review of European research on consumer response to nutrition information on food labels. J. Public Health.

[40] Croker H., Packer J., Russell S.J., Stansfield C., Viner R.M. (2020). Front of pack nutritional labelling schemes: A systematic review and meta-analysis of recent evidence relating to objectively measured consumption and purchasing. J. Hum. Nutr. Diet..

[41] (2021). World Health Organization Coronavirus Disease (COVID-19) Pandemic. https://www.who.int/emergencies/diseases/novel-coronavirus-2019.

[42] Motti M.L., Tafuri D., Donini L., Masucci M.T., De Falco V., Mazzeo F. (2022). The Role of Nutrients in Prevention, Treatment and Post-Coronavirus Disease-2019 (COVID-19). Nutrients.

[43] Kumar P., Kumar M., Bedi O., Gupta M., Kumar S., Jaiswal G., Rahi V., Yedke N.G., Bijalwan A., Sharma S. (2021). Role of vitamins and minerals as immunity boosters in COVID-19. Inflammopharmacology.

[44] Im J.H., Je Y.S., Baek J., Chung M.-H., Kwon H.Y., Lee J.-S. (2020). Nutritional status of patients with COVID-19. Int. J. Infect. Dis..

[45] Pedrosa L.F., Barros A.N., Leite-Lais L. (2021). Nutritional risk of vitamin D, vitamin C, zinc, and selenium deficiency on risk and clinical outcomes of COVID-19: A narrative review. Clin. Nutr. ESPEN.

[46] Verschelden G., Noeparast M., Noparast M., Goossens M.C., Lauwers M., Cotton F., Michel C., Goyvaerts C., Hites M. (2021). Plasma zinc status and hyperinflammatory syndrome in hospitalized COVID-19 patients: An observational study. Int. Immunopharmacol..

[47] Elham A.S., Azam K., Azam J., Mostafa L., Nasrin B., Marzieh N. (2021). Serum vitamin D, calcium, and zinc levels in patients with COVID-19. Clin. Nutr. ESPEN.

[48] Younesian O., Khodabakhshi B., Abdolahi N., Norouzi A., Behnampour N., Hosseinzadeh S., Alarzi S.S.H., Joshaghani H. (2021). Decreased Serum Selenium Levels of COVID-19 Patients in Comparison with Healthy Individuals. Biol. Trace Element Res..

[49] Majeed M., Nagabhushanam K., Gowda S., Mundkur L. (2021). An exploratory study of selenium status in healthy individuals and in patients with COVID-19 in a south Indian population: The case for adequate selenium status. Nutrition.

[50] Lai Y.-J., Chang H.-S., Yang Y.-P., Lin T.-W., Lai W.-Y., Lin Y.-Y., Chang C.-C. (2021). The role of micronutrient and immunomodulation effect in the vaccine era of COVID-19. J. Chin. Med Assoc..

