# Supplementary material for: The Perception of Minerals and Their Prevalence in Fortified Foods and Supplements in Japan
Source: Nutrients. 2022 Jun 22;14(13):2586. doi: 10.3390/nu14132586 (PMC9268031; doi:10.3390/nu14132586)
Supplement: Supplementary file 1 [file nutrients-14-02586-s001.zip › nutrients-1766228-supplementary.pdf]

Questionnaire

Q1-1. How often do you consume a well-balanced diet?

A well-balanced diet is having a meal consisting of the following three dishes at least twice a day.<br>Grain dish (rice, bread, noodles, etc.)<br>Main dish (dishes mainly made of meat, fish, eggs, soybeans, soy products, etc.)<br>Side dish (dishes mainly made of vegetables, seaweed, mushrooms, etc.)

1. Almost every day
2. 4-5 days a week
3. 2-3 days a week
4. Rarely

Q1-2. What is(are) the reason(s) you cannot consume a well-balanced diet? (Multiple answers)

1. I have no time to spare.
2. I do not have enough money.
3. There are many opportunities to eat out.
4. I often buy lunch boxes and prepared dishes.
5. Others

Q2-1. Do you use mineral-fortified food?

"Mineral-fortified food" are foods that are sold as a regular food, but are labeled as "added Y xx mg", "Z xx% fortified", etc.

1. I actively use it.
2. I use it occasionally.
3. I have never used it.
4. I do not care.

Q2-2. What is(are) the reason(s) for not using mineral-fortified food? (Multiple answers)

1. I feel that I can take enough with my usual diet.
2. No mineral-fortified foods that I want to consume.
3. I use other products such as supplements.
4. It is expensive to buy.
5. I do not want to spend money on fortified foods.
6. I have bought it, but it was not delicious.
7. I have bought it, but it did not suit my health.
8. Others

Q3-1. Do you use mineral supplement?

There is no definition of supplement in Japan. In this survey, capsules, tablets, and other forms that containing condensed specific ingredients are defined as supplement.

1. I currently use mineral supplement.
2. I currently use non-mineral supplement.
3. I used to use it, but not now.
3. I have never used it.

Q3-2. What is(are) the reason(s) for not using mineral supplement? (Multiple answers)

1. I feel that I can take enough with my usual diet.
2. No mineral supplements that I want to consume.
3. I use other products such as fortified foods.
4. It is expensive to buy.
5. I do not want to spend money on supplements.
6. I have bought it but it did not suit my health.
7. Others

Q4. Do you know "Foods with Nutrient Function Claims."?

1. I do not know about it.
2. I know it, but I do not use it.
3. I currently use it.

Q5. How much do you know about each mineral?

	I understand its role in the body well.	I understand somewhat.	I have only heard about it.	I do not know.
Sodium				
Potassium				
Calcium				
Magnesium				
Phosphorus				
Zinc				
Iron				
Copper				
Manganese				
Iodine				
Selenium				
Chromium				
Molybdenum				

Q6. How do you think of your mineral intake status?

	Enough	Insufficient	I do not know
Sodium			
Potassium			
Calcium			
Magnesium			
Phosphorus			
Zinc			
Iron			
Copper			
Manganese			
Iodine			
Selenium			
Chromium			
Molybdenum			

Q7. Choose the minerals that you consciously ingest.

Sodium	
Potassium	
Calcium	
Magnesium	
Phosphorus	
Zinc	
Iron	
Copper	
Manganese	
Iodine	
Selenium	
Chromium	
Molybdenum	
None	

(Following questions are asked to only mineral-fortified food/mineral supplement users.)

Q8. What is(are) the purpose(s) to use mineral-fortified food/mineral supplement? (Multiple answers)

1. Maintenance of health
2. Supplementation of nutrients
3. Beauty benefits
4. Weight loss
5. Building muscle
6. Improvements to health
7. Prevention of diseases
8. Treatment of diseases
9. Improvement of immune function / prevention of infectious diseases
10. Other

Q9. Do you know "the Dietary Reference Intakes for Japanese"?

"The Dietary Reference Intakes for Japanese" proposes reference values for the intake of energy and nutrient to promote and maintain heath, and to prevent lifestyle-related diseases.

1. I know about it and I use it.
2. I know about it, but I do not use it.
3. I have only heard about it.
4. I do not know about it.

Q10. Do you check mineral content labeled on fortified food/supplement packages?

1. I always check it.
2. I sometimes check it.
3. I do not check it.
4. I did not know that it was labeled.

Q11. Do you know the amount of each mineral that you take?

1. I know the intake amount.
2. I don't know the intake amount.
3. I do not care about it.

Q12. Do you use application programs that tells calories and nutrient contents by taking a photo of your meal?

1. I currently use them.
2. I used to use them, but not now.
3. I have never used them.
